# Design and evaluation of a co-produced social media campaign to promote aquatic safety in Queensland national parks

**DOI:** 10.1093/heapro/daaf181

**Published:** 2025-10-30

**Authors:** Samuel Cornell, Timothy Piatkowski, Robert W Brander, Amy E Peden

**Affiliations:** School of Population Health, Faculty of Medicine and Health, Level 5, Health Translation Hub, UNSW Sydney, Kensington, NSW 2052, Australia; School of Applied Psychology and Griffith Centre for Mental Health, Level 4 Psychology Building (M24), Griffith University, Messines Ridge Road, Mt Gravatt, QLD 4122, Australia; School of Biological, Earth and Environmental Sciences, Biological Sciences Building (D26) , UNSW Sydney, Kensington, NSW 2052, Australia; School of Population Health, Faculty of Medicine and Health, Level 5, Health Translation Hub, UNSW Sydney, Kensington, NSW 2052, Australia; College of Public Health, Medical and Veterinary Sciences, James Cook University, 1 James Cook Drive, Douglas, QLD 4811, Australia

**Keywords:** social media, national parks, Australia, land managers, risk-taking, outdoor recreation, drowning, injury, risk communication, safety promotion, health promotion

## Abstract

Social media increasingly shapes how visitors engage with aquatic locations in national parks, where risky behaviours contribute to drowning and injury risk. Selfies and photography with mobile phones have been implicated in fatal and non-fatal injury, due to distraction leading to a loss of awareness of a person’s surroundings. We partnered with Queensland Parks and Wildlife Service (QPWS) to co-produce and evaluate a targeted Instagram water safety campaign at an injury hotspot, with specific focus on campaign design, audience engagement, message recall, and user perceptions. Campaign development was informed by social media user and influencer interviews, surveys with social media users, frontline ranger observations, and patterns of visitor behaviour on social media. Between January and February 2024, Instagram posts highlighting site-specific risks were disseminated. The campaign generated strong engagement: over 4000 link clicks, more than 100 shares, and 254 post saves. Content analysis showed polarized user responses: 20% found the messaging informative and useful, while another 20% found it patronizing. In-person surveys at the site (*n* = 50) found that 32% of visitors reported social media influenced their decision to visit. Separately, a third (32%) of participants recalled at least one of the campaign’s safety messages, with Post B being the most recalled. The findings suggest that social media can be a low-cost and impactful tool for aquatic risk communication at high-visitation sites. Future work should focus on refining tone, testing co-design approaches with target audiences, and exploring the potential role of influencer partnerships to enhance reach and resonance.

Contribution to Health Promotion

- Social media affects how people behave in national parks, and can lead them to take more risks, especially around water.
- This study shows that well-designed, co-produced social media campaigns can help keep people safer by raising awareness of dangers.
- Working with park staff and social media influencers may help get safety messages to people who are more likely to take risks.
- Social media is a cheap and impactful way to promote safety and prevent injuries and deaths.
- Future work should focus on improving how safety messages are shared online and potentially partner with influencers so that messages have peer approval.

## INTRODUCTION

The integration of social media and smartphone technology has significantly altered traveller behaviour (Zeng and Gerritsen 2014). The ‘selfie gaze’ and the desire to share tourism experiences online, influences how visitors select and engage with destinations (Magasic 2016). As a result, ‘selfie-tourism’ and social media driven tourism (Munar and Jacobsen 2014, Magasic 2016, Elshaer *et al*. 2022) are impacting visitation to national parks, which have become popular natural backdrops for those seeking to elevate their social media presence with compelling images, earning ‘likes’ and ‘shares’. Tourists are increasingly drawn to places that promise visually stunning experiences, capable of generating significant social media engagement (Cornell *et al*. 2024b).

This shift has been fuelled further by travel influencers, whose large followings amplify the attractiveness of scenic spots such as those found in national parks (Harrigan *et al*. 2021, Manthiou *et al*. 2024). Influencers’ content shapes destination choice and visitor behaviour, sometimes promoting risky activities for engagement (Bozzi 2020) including at aquatic locations. These environments possess unique, and sometimes unpredictable, hazards that visitors may overlook, such as large and irregular waves, strong currents, sudden water depth changes, and slippery surfaces. The rise of social media promoting these sites underscores the need for pre-emptive safety interventions.

There have been multiple instances of incidents leading to fatalities as tourists attempt potential dangerous feats for social media all over the world (Cornell *et al*. 2023). For example, cliff side poses and seaside photographs have led to fatal falls and drownings (Cornell *et al*. 2023, Cornell *et al*. 2024b, Cornell and Peden 2024). At aquatic locations, the influence of social media has been linked to both fatal and non-fatal drowning. For example, in Australia, a 20-year-old man drowned at No.16 Beach on the Mornington Peninsula after being drawn there by TikTok videos showcasing its beauty, with locals blaming social media for encouraging visits to dangerous unpatrolled surf beaches (Valentish and Hone 2024). In response to such incidents, water safety alliances have launched campaigns directly addressing the influence of TikTok, Instagram and Facebook on risky aquatic behaviour, highlighting how curated online imagery can obscure real dangers (Lindsay 2025). The presence of tourists unfamiliar with responsible travel practices (e.g. checking weather and tidal conditions, or warning signs, wearing appropriate clothing) heightens the need for targeted educational content accompanied by direct warnings via technology to manage the impact of this new wave of tourism.

As this new tourist (Brennan 2023) is driven by social media to visit picturesque destinations, we argue it may be prudent to ‘meet people where they are’ and engage them via social media in order to communicate risk and safety information about aquatic locations (Cornell *et al*. 2024c). Strengthening this call, Australian research has shown that among parks users, social media, rather than an official Parks website, are more frequently visited for site and/or safety information (Cornell *et al*. 2025).

This trend is mirrored internationally, where social media use has been shown to influence park visitation patterns and even correlate with increased search and rescue incidents in US national parks (Lu *et al*. 2021, Wichman 2024). Considering this, social media may be seen as both a boon and a bane to health promotion and safety. On the one hand, social media has driven an increase in misinformation, such as in the realm of vaccine hesitancy (Dodd *et al*. 2022) and ideas of ‘wellbeing’ that are unsubstantiated or even harmful (Gibbs and Piatkowski 2023). However, on the other hand, social media may be a useful avenue for authorities to combat such misinformation, capitalizing on the reach that social media has amongst a captive audience (Taba *et al*. 2023, Cornell *et al*. 2024). Given the influence of social media and the credibility attributed to influencers, leveraging trusted content creators can enhance message resonance and trustworthiness (Lou and Yuan 2019).

Conventional park safety messaging—such as signage, printed materials, and ranger-led education—has proven effective for some audiences (Saunders *et al*. 2019), but lacks the real-time, shareable nature of social media content. Social media campaigns, particularly those employing influencer partnerships or interactive features, have the potential to reach a wider audience and reinforce behavioural norms in a more engaging manner. However, their effectiveness relative to traditional methods remains underexplored, making this study an important step in evaluating the role of digital safety interventions in national parks (Weiler *et al*. 2021).

Studies have suggested that social media has utility for behaviour change and modifying health harming behaviours beyond simply raising awareness (Evans *et al*. 2022, Ghahramani *et al*. 2022). Social media platforms allow users to participate and create content, engaging them within their social networks and fostering a sense of connection to the message. Social media strategies can create a sense of widespread support for certain behaviours (Evans *et al*. 2022, Merchant *et al*. 2021). In both positive and negative ways, social media has created widespread support for specific behaviours. For example, viral ‘challenges’ and influencer content have normalized dangerously jumping into bodies of water (‘tombstoning’) (Moran 2014), and entering restricted aquatic zones for photographs. Conversely, social media has also been harnessed to encourage positive health behaviours such as handwashing during COVID-19 (Zhang *et al*. 2020). Harnessing social media for behaviour change involves utilizing the interactive affordances of social platforms to encourage people to adopt new behaviours or maintain existing ones (Burgess *et al*. 2017).

Social media can become a powerful health promotion lever, but only if health promotion practitioners create engaging content, thus encouraging the target audience to ‘buy in’ (Gough *et al*. 2017). The interactive nature of social media platforms allows for direct participant interaction through comments, messages, and live sessions, facilitating support and encouragement for healthy behaviours. Not only does this intervention style enable targeted and expedient messaging to be sent to the people of interest, but the approach is cost-effective compared to traditional media and can reach broader and more diverse audiences, including hard-to-reach populations, thereby enhancing the overall impact of health promotion efforts (Korda and Itani 2013).

In this study, we focus specifically on aquatic safety in national parks, recognizing that aquatic hazards such as drowning and injury are among the most serious risks in these environments (Girasek *et al*. 2016).

### Theoretical framing

The theory of planned behaviour (TPB) (Bosnjak *et al*. 2020) provides a useful framework for understanding how social media campaigns may influence safety-related decision-making. TPB posits that behaviour is shaped by attitudes, subjective norms, and perceived behavioural control. In the context of this study, attitudes towards risk-taking, the influence of social media norms (e.g. peer behaviour and influencer promotion of certain activities), and perceived ability to assess risk and avoid hazards are key factors in determining visitor behaviour at aquatic sites.

The TPB (Bosnjak *et al*. 2020) is particularly relevant in addressing behaviours such as ignoring warning signs at aquatic locations and engaging in risky activities in natural environments, which can lead to fatalities and injuries such as drowning (Cornell *et al*. 2024a). In the context of preventing accidental harm in natural environments, the TPB is a useful framework for designing health behaviour campaigns (Darker *et al*. 2010 , Juraskova *et al*. 2012, Kothe *et al*. 2012). More recently, the TPB has been extended to explore social media-based interventions (Li *et al*. 2022). Zheng *et al*. (2022) used TPB to highlight the significant impact of social media on revisit intention, suggesting strategies for enhancing tourist satisfaction and encouraging repeat visits through targeted social media engagement.

Operating on a similar premise, a social media communication campaign to discourage risky health behaviours could promote safety among tourists. Specifically relevant to the TPB, social media campaigns can be used to change attitudes by highlighting the dangers of ignoring warning signs and sharing real-life stories of incidents to evoke emotional responses. Subjective norms can be influenced by showcasing respected community figures, representatives of authoritative bodies, and peers who advocate for safety and caution in natural settings. Perceived behavioural control can be enhanced by providing practical tips and resources on how to stay safe, such as by providing information on safe swimming areas and information on identifying and responding to hazards. By addressing these components, TPB-based interventions can effectively reduce risky behaviours and promote safer practices in the natural environment.

In this study, TPB was applied to assess whether social media safety messaging could influence visitors’ attitudes towards risk-taking, shape perceived social norms around safe behaviour in national parks, and enhance their perceived behavioural control in recognizing and avoiding hazards. By structuring campaign messages around these behavioural determinants, the study aimed to evaluate whether targeted risk communication could lead to safer decision-making at aquatic sites.

### Aims

As increasing numbers of visitors are drawn to aquatic locations in national parks by social media (Wichman 2024), this study aimed to design, implement, and evaluate a co-produced social media campaign promoting aquatic safety in a Queensland national park. These aims were addressed through three sequential phases:

1. Phase 1: Formative research and campaign development—To conduct qualitative and quantitative research to inform message content, and to collaborate with land managers to design the social media campaign.
2. Phase 2: Campaign implementation—To deliver targeted messages across social media platforms and related communication channels.
3. Phase 3: Campaign evaluation—To assess campaign delivery and its impact on knowledge, user engagement, message recall, and audience perceptions.

## MATERIALS AND METHODS

### Design and ethics

This study employed a mixed-methods co-production approach. Co-production was defined as a collaborative process (Vargas *et al*. 2022) in which researchers and land managers jointly developed the campaign content and strategy, drawing on previous research that included interviews and surveys with influencers and social media users. The participatory approach incorporated insights from social media users, travel influencers, and QPWS staff to ensure the messaging aligned with audience expectations and risk communication best practices (De Rosis *et al*. 2020, River *et al*. 2023). A campaign was then developed using this co-production and participatory framework, informed through previous qualitative interviews with social media users and travel influencers (Cornell *et al*. 2024b).

**Figure 1.**
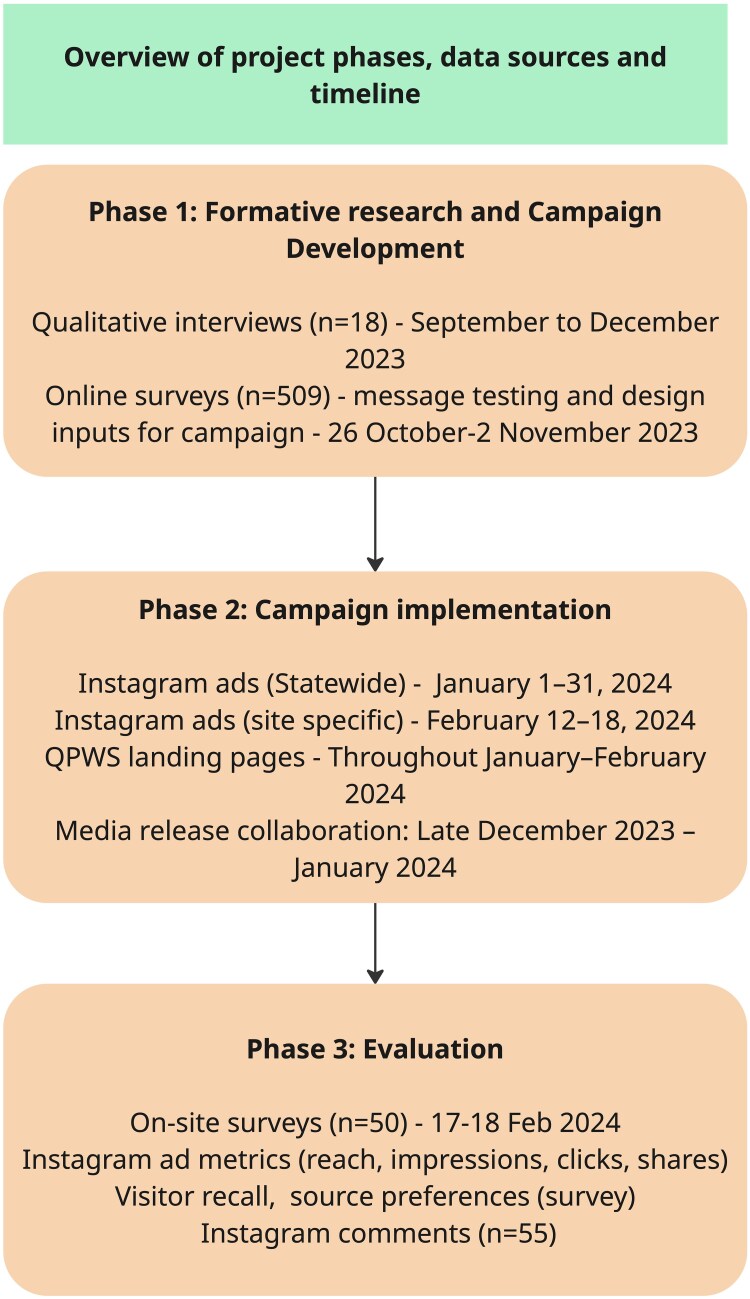
Overview of project phases, data sources, and timeline for Phase 1, Phase 2, and Phase 3 of the social media risk communication campaign.

This paper describes three phases of the study (Fig. 1). Phase 1 involved formative research and the co-production of a social media risk communication campaign, including qualitative interviews with influencers and an online survey of social media users to inform message content and design. Phase 2 focused on campaign implementation across selected social media channels and associated communication platforms. Phase 3 evaluated the campaign by triangulating delivery metrics with quantitative and qualitative analyses of user experiences. Ethics approval was granted by the UNSW Sydney Research Ethics Committee (Project ID 4989). Participants were provided with an information sheet and gave verbal consent only, as approved by the UNSW Human Research Ethics Advisory Panel.

### Phase 1: formative research and campaign co-production

Campaign messaging was co-developed by QPWS and the UNSW Sydney Beach Safety Research Group, grounded in formative research that included a national survey of 509 social media users (Cornell and Peden 2025) and interviews with 18 travel and adventure influencers. This formative research directly informed campaign strategy and design, particularly regarding platform choice, content tone, and message format (Supplementary File S1).

Survey results identified Instagram as the most influential platform for planning park visits, especially among younger users (defined in this study as 18–35 years). Participants preferred safety information delivered in a relatable, informal tone through the same social media channels where they encountered aspirational travel content (‘travel-spo’). Many indicated they wanted this content to come directly from land managers, reinforcing credibility and authenticity.

Interviews with influencers revealed that while many were aware of risk-taking behaviours in park settings, they typically did not view themselves as responsible for how followers interpreted or replicated their content. Several expressed the view that land managers should be doing more to proactively communicate safety expectations and mitigate the potential downstream effects of influencer posts. This further reinforced the need for a campaign that could provide official, pre-emptive messaging within the same digital spaces where risky content circulated.

To align with these insights, the campaign was designed to funnel users from Instagram ads and organic posts to tailored landing pages on the QPWS website. These pages were written in a tone that mirrored the target audience's online language—conversational, visually framed, and culturally aligned with social media norms. Content included realistic safety framing, respectful humour, and practical tips for both safe behaviour and authentic content creation.

As part of the Phase 1 survey, participants were asked to select the safety message they found most compelling from a list of candidate options. These phrases were developed through a co-production process involving Queensland Parks and Wildlife Service (QPWS) staff, informed by previous water safety campaigns, expert advice, and formative research (interviews with influencers, land managers, and surveys of social media users). The most frequently selected message was ‘Make sure time in nature doesn’t come at a cost’ (*n* = 104, 20.4%), followed by ‘Take our signs as a sign to stay safe’ (*n* = 90, 17.7%) and ‘Don’t let your life slip away’ (*n* = 89, 17.5%). Other popular choices included ‘Even inviting, natural places can be life-threatening’ (*n* = 85, 16.7%) and ‘For parks’ sake, stay safe’ (*n* = 61, 12.0%). Less frequently selected messages included ‘What you see isn’t what you get’ (*n* = 37, 7.3%) and ‘For the love of parks, take care in and around water’ (*n* = 30, 5.9%). A small proportion of respondents selected ‘Other’ and provided their own wording (*n* = 13, 2.6%). These responses did not propose alternative slogans so much as express cynicism or frustration. Common themes included appeals to ‘common sense’ (‘use common sense people’, ‘choices have consequences’), dismissal of responsibility (‘none’, ‘not my problem’), or blaming risky individuals themselves (‘idiots’, ‘survival of the fittest’). A small number mentioned sources of influence (friends, family, celebrities, Aboriginal Australians) or practical measures such as signage.

Overall, messages that framed nature-related risk in direct, emotive, or consequence-focused language were most compelling to participants. In contrast, more abstract or metaphorical phrasing received lower endorsement. These preferences directly informed the final message set used in the campaign, with the most highly rated messages integrated across social media posts, landing pages, and visual assets.

### Campaign development

The campaign was developed using a participatory and co-production approach (Vargas *et al*. 2022). These insights shaped the messaging strategy and creative materials, ensuring alignment with audience expectations and risk communication best practices. Key themes identified from consultations influenced the tone, format, and dissemination of content to maximise engagement and message retention. The lead author and the main contact at QPWS determined the best course of action was to create a campaign based on current social media trends, such as the ‘Point of View’ (POV) aspect and to play into travel influencer trends such as ‘chasing waterfalls’ and using Gen X/Millennial social media language such as ‘inspo’.

The campaign messaging was explicitly designed to align with TPB constructs (Ajzen 1991). It aimed to explicitly integrate the three constructs of TPB (attitudes, subjective norms, perceived behavioural control).

1. Attitudes: Campaign posts highlighted real-life consequences of risk behaviours, such as injury or rescue incidents, aiming to change visitor perceptions of risk-taking around aquatic hazards.
2. Subjective norms: Messages featured endorsements by authoritative figures (e.g. park rangers) and popular influencers, reinforcing community-approved safe behaviours.
3. Perceived behavioural control: Posts provided clear and actionable safety tips, empowering visitors with practical knowledge to avoid risks (e.g. recognizing hazardous zones, understanding signage)

### Campaign materials

Based on this approach, a suite of digital communication materials was created to target social media users engaging with national park content. The primary components included:

1. State-wide advertisements: Designed to appeal broadly to potential visitors, incorporating engaging visuals and relatable narratives.
2. Site-specific advertisements: Tailored messaging for particular locations, emphasizing hazard awareness and site-specific safety information.
3. Landing pages: Hosted on the QPWS website, providing expanded safety guidance and practical recommendations for visitors.
4. Organic social media content: Posts published directly through QPWS accounts to reinforce campaign messaging.

### Phase 2: campaign implementation

**Figure 2.**
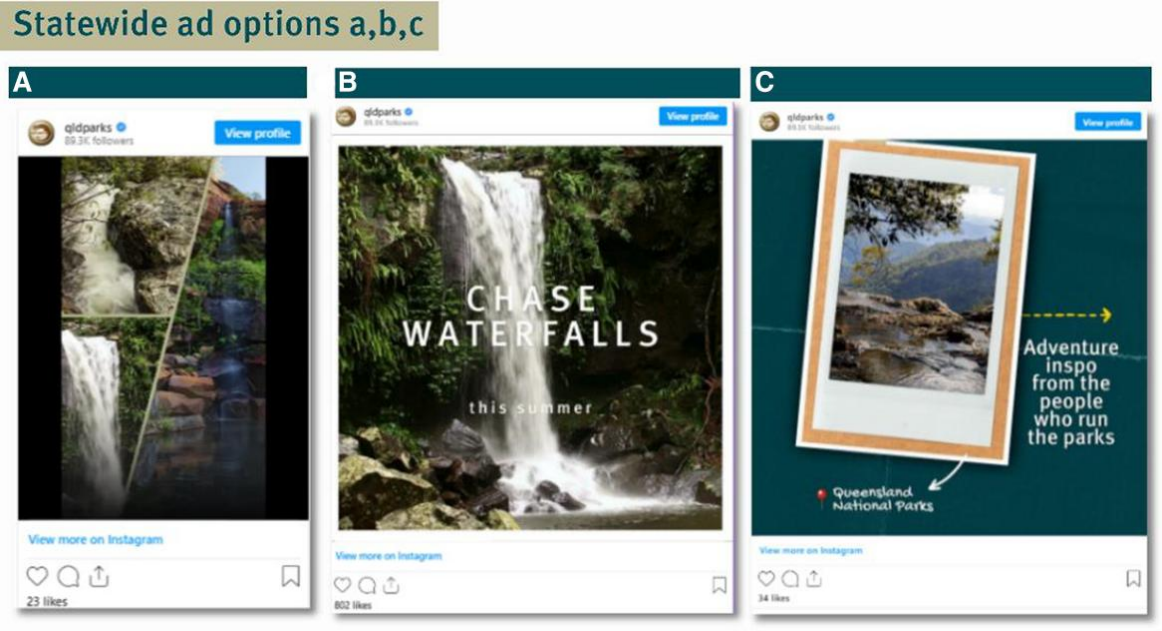
Panel showing statewide ads (a, b, and c) displayed to users on Instagram and Facebook.

The campaign was executed via a combination of paid and organic social media strategies across state-wide and site-specific advertisements. In terms of state-wide advertisements (Fig. 2), from 1 January to 31 January 2024, four paid Instagram ads targeted Australian users aged 18–35 with interests in travel, outdoor creators, national parks, and Queensland tourism. The ad placements appeared in Instagram feeds, explore pages, search results, and profile feeds. The creative formats included carousels with still images and GIFs (animated images), as well as video-based Reels, designed to leverage the popular ‘POV’ trend.

**Figure 3.**
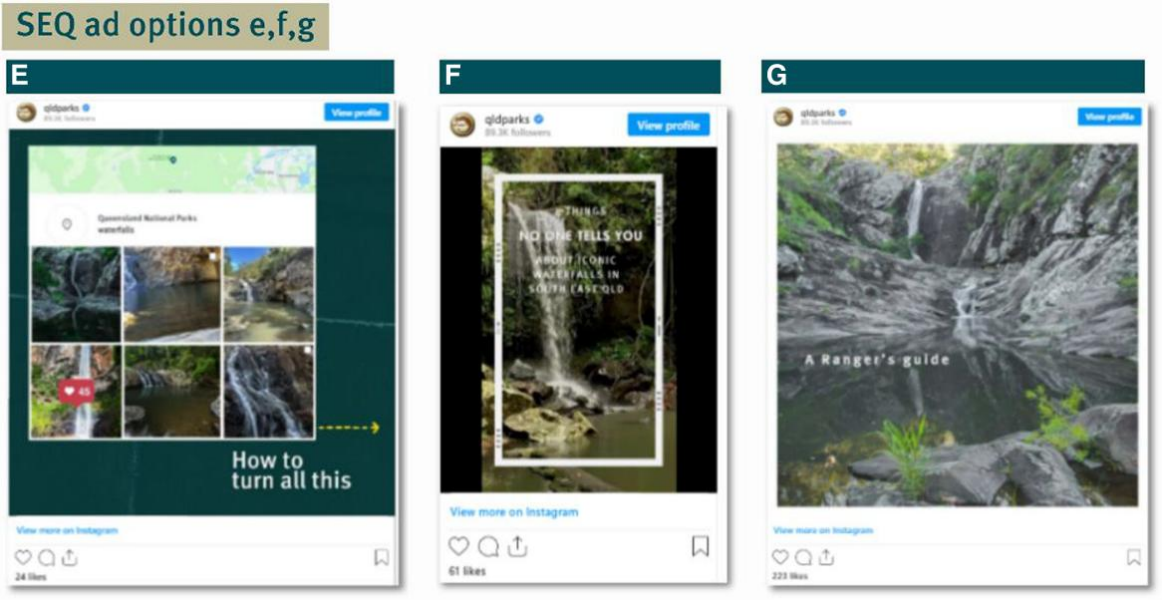
Panel showing Southeast Queensland (SEQ) campaign ads (e, f, g); ad d was excluded from recall testing due to low reach.

The site-specific advertisements (Fig. 3) ran from 12 February 12 to 18 February 2024 with targeted Instagram ads geolocated to Curtis Falls, Tamborine National Park in Queensland. These ads contained more information including restricted access area and waterfall safety advice, realities of how busy these areas can be and other location recommendations to disperse visitor traffic to other national park locations. QPWS promoted the campaign through its social media channels and email newsletters, supported by a media release developed in collaboration with UNSW Sydney Beach Safety Research Group.

#### Landing pages

Two campaign landing pages, one statewide and one specific to South East Queensland (SEQ), were hosted on the QPWS website and served as key hubs for behavioural messaging. Instagram ads directed users to the appropriate page based on geographic targeting (Supplementary File S2).

The statewide page addressed general safety and expectation management, using relatable language and humour informed by formative research with younger social media users. It focused on content realism, dynamic environmental conditions, and responsible sharing practices (e.g. ‘catphished by a park’). The SEQ page focused on site-specific risks related to waterfalls, including overcrowding, unstable terrain, and restricted areas. It also promoted alternative park locations to help reduce visitor pressure.

Both pages integrated TPB principles:

1. ‘Attitudes’: Highlighting real risks and consequences
2. ‘Subjective norms’: Promoting safe, authentic content-sharing
3. ‘Perceived behavioural control’: Providing clear tips and resources for safe visitation

Although not formally evaluated through analytics, the pages served as trusted touchpoints for reinforcing campaign messaging.

### Phase 3: campaign evaluation

The first part of the evaluation involved field surveys conducted in-person by the lead author from Saturday 17th to Sunday 18th February 2024 at Curtis Falls in Tamborine National Park. The lead author set up a survey collection site at the entrance to the sole trail that led down to the waterfall (the main tourist attraction at the site). Data collection was originally planned to take place at Cedar Creek Falls on the same dates. However, due to severe storms, this site was closed over the study period, so the alternative site of Curtis Falls was chosen, which is a 5.7 km drive away from the former and is also within Tamborine National Park.

#### Participants and recruitment

Participants were eligible to take part if they were aged 18 years or older, spoke and read English and were visiting Tamborine National Park during the data collection period. Potential participants were approached on site by the researcher and asked if they would like to participate in a survey. Participants were provided information on the study in the form of a participation information sheet and consent form and asked to provide verbal consent if they would like to take part.

Although Curtis Falls is not an official swimming site, it features frequent visitor interactions with aquatic hazards such as slippery surfaces and waterfalls. QPWS Rangers are aware of incidents and visitors climbing barriers to approach hazardous areas for photographs or social media content. This justified Curtis Falls as an appropriate site for evaluating a targeted aquatic risk communication campaign.

#### Survey measures

Demographic survey items included age, gender, whether born in Australia, and main language spoken at home. Participants were able to choose whether they were a ‘local’ or ‘tourist’ (self-determined) to the area. The survey assessed aspects of social media-driven national parks visitation in Queensland and aimed to understand the type of content that social media users wished to see regarding risk communication. Furthermore, comments provided by social media users directly on the communication materials were analysed to gain insights into user perceptions and preferences, complementing the survey data on social media-driven national parks visitation and content preferences.

### Analysis

#### Statistical analysis

Preliminary analysis of quantitative data was performed using Microsoft Excel, with robust analyses conducted using R. Descriptive statistics were generated for demographic characteristics of the analysed sample. To determine the significance of the observed differences in visitation to national parks attributed to social media across demographics, a Pearson’s chi-square test for independence was conducted (*P* < .05). Where multiple categories within a variable were analysed a modified Bonferroni correction was applied (Keppel and Wickens 2004). Ad recall was assessed as prompted recognition, consistent with methods used in mass media campaign evaluations (Casten *et al*. 2022).

#### Content analysis

Instagram comments on the campaign posts were analysed using content analysis methodology (Downe-Wamboldt 1992, Cornell *et al*. 2023). A combined inductive–deductive approach was employed. First, inductive coding was used to identify recurring categories and audience perceptions directly from the data (Proudfoot 2023). These emergent themes were then organized and refined using a deductive framework developed prior to coding, informed by risk communication literature and the TPB. This framework focused on audience attitudes, perceptions of norms, and message reception, and is summarized in Supplementary File S3. Comments were coded across the following categories: agreement with the message, frustration or resistance, influencer criticism and authenticity, photography and artistic expression, perceptions of park management or visitor experience, and criticism of post tone. Coders could apply multiple categories per comment. Two researchers independently coded all comments using the shared framework and discrepancies were resolved through discussion until consensus was reached, fitting with previous work (Piatkowski *et al*. 2021).

## RESULTS

This section presents the findings from the evaluation of the co-produced social media campaign, including social media engagement metrics, landing page traffic, in-person survey results, and a content analysis of user comments.

### Campaign evaluation: metrics

Overall, the social media communication campaign reached approximately 962 000 social media users, with 83 800 engagements and 1.9 million impressions. There were 254 total saves of the posts, with 4000 link clicks that took the user through to the landing pages which presented more risk information on the sites. The posts were shared a total of 102 times, and 55 comments were left by users across the communication campaign. According to Meta, ∼16 000 users would have been able to recall the ads (Table 1). QPWS also increased their social media following by more than 1000 users during the Phase 2.

**Table 1.** Social media metrics, including reach and engagements, of the communication campaign materials.

Campaign material	Reach/views^a^	Link clicks (to land pages)	Engagements^b^	Ad recall lift	Comments	Saves	Cost [Australian dollars (AUD)]
General ads (statewide)	316k	3.1k	42k	N/A	18	201	$2390
Southeast Queensland targeted ads	293k	470	18.9k	7.4k	N/A	25	$2215
Cairns (Josephine falls) targeted ads	255k	490	21.8k	9k	N/A	14	$2362
Organic content	97.9k	646	1.5k	N/A	57	N/A	N/A
Landing (web) pages supporting material	4155	N/A	N/A	N/A	N/A	N/A	N/A
Total	1 924 420	4706	84 200	16 400	55	240	$6967

^a^Refers to the total number of unique social media users exposed to the campaign content. A higher reach indicates greater overall awareness potential for the safety messages (Lou and Yuan 2019). ^b^Includes interactions such as likes, comments, shares, and saves. High engagement rates suggest active user interest, deeper cognitive processing, and potential behavioural influence from the campaign messages (Gough *et al*. 2017).

### Landing page engagement

Web traffic analytics showed that the campaign’s landing pages attracted substantial user engagement. The general statewide page received 3100 views, while the SEQ-specific page had 565 views. These figures reflect meaningful traffic driven by both paid and organic posts, and suggest that Instagram ads effectively funnelled users towards safety information hosted on official platforms.

### Campaign evaluation: survey findings

These results assess whether social media influenced participants’ decision to visit.

The in-person surveys had a total of 50 respondents. In total, 26 (52%) participants identified as female, 25 (50%) were born in Australia, 36 (72%) considered themselves to be tourists to the area, and 14 considered themselves locals. Sixty four percent (*n* = 32) of participants spoke English at home with 18 speaking a language other than English. Half (*n* = 25, 50%) said they visited national parks a ‘few times per year’ (Table 2).

**Table 2.** Sample characteristics and statistical analysis of social media–attributed visitation to the national park.

	Total number of sample (%)	Social media attributed visitation to the national park		χ² (df)	*P*-value
		Yes	No		
Variable	*N* (%)	*N* (%)	*N* (%)		
**Total**	50 (100)	16 (32)	34 (68)		
**Age group**				1.82 (3)	0.61
18–24	13 (26)	4 (31)	9 (69)		
25–34	19 (38)	8 (42)	11 (58)		
35–44	12 (24)	3 (25)	9 (75)		
45–54	6 (12)	1 (17)	5 (83)		
**Gender**				0.01 (1)	0.91
Man/male	24 (48)	7 (29)	17 (71)		
Woman/female	26 (52)	9 (35)	17 (65)		
**Location born**				2.30 (1)	0.13
Australia	25 (50)	5 (20)	20 (80)		
Overseas	25 (50)	11 (44)	14 (56)		
**Local or visitor^a^**				0.00 (1)	1.00
Local	14 (28)	4 (71)	10 (29)		
Visitor	36 (72)	12 (33)	24 (67)		
**Main language spoken at home**				1.21 (1)	0.27
English	34 (68)	8 (24)	24 (76)		
Other	16 (32)	8 (44)	10 (56)		
**Most used social media platform**				3.92 (2)	0.14
Instagram	28 (56)	9 (32)	19 (68)		
Facebook	14 (28)	3 (21)	11 (79)		
Other	8 (16)	5	3		
**How often do you visit national parks?**				1.09 (3)	0.78
Few times per year	25 (50)	7 (28)	18 (72)		
Monthly	13 (26)	4 (31)	9 (69)		
Weekly	6 (12)	3 (50)	3 (50)		
Rarely	6 (12)	2 (33)	4 (67)		
**Do you research national parks before you visit them?**				0.77 (1)	0.38
Yes	42 (84)	15 (36)	27 (64)		
No	8 (16)	1 (12)	7 (88)		
**Do you visit specifically to take photographs?**				8.48 (1)	0.004*
Yes	11 (22)	8 (72)	3 (27)		
No	39 (78)	8 (20)	31 (80)		
**Frequency of social media for reliance for visiting national parks**				9.17 (3)	0.027*
Often	20 (40)	11 (55)	9 (45)		
Occasionally	17 (34)	4 (23)	13 (76)		
Rarely	8 (16)	1 (12)	7 (87)		
Never	5 (10)	0 (0)	5 (100)		
**Have you ever taken risks to get social media content?**				0.24 (1)	0.62
Yes	9 (18)	4 (44)	5 (56)		
No	41 (82)	12 (29)	29 (71)		
**Would other people consider your social media content to be risky?**				0.00 (1)	0.96
Yes	8 (16)	2 (25)	6 (75)		
No	42 (84)	14 (67)	28 (33)		

^a^Survey respondents self-selected whether they were a local or visitor. *Indicates statistically significant result.

In-person surveys at Curtis Falls (*n* = 50) found that 32% (*n* = 16) of visitors reported social media influenced their decision to visit the site. Age group differences in visitation influenced by social media were not statistically significant (*P* = .61), and no significant associations were found by gender (*P* = .91), country of birth (*P* = .13), or language spoken at home (*P* = .27). Two variables did show significant relationships: visitors who reported visiting specifically to take photographs were substantially more likely to attribute their visit to social media (72% vs. 20%; χ² = 8.48, *P* = .004), and those who frequently used social media when planning park visits were also more likely to attribute their visitation to social media use (55% ‘often’ vs. 0% ‘never’; χ² = 9.17, *P* = .027). No significant associations were found for self-reported risk-taking behaviours (*P* = .62) or whether others perceived participants’ social media content as risky (*P* = .96) (Table 2).

Survey respondents were asked which mediums they prefer to receive risk-related information on when visiting national parks. The most commonly selected responses were on-site signage (*n* = 32; 64%), followed by social media posts from an official national parks account (*n* = 22; 44%), and general risk information on national parks websites (*n* = 18; 36%). Other selections included; visual examples of risks (photos, infographics, videos etc.) online (*n* = 16), slogan-based messaging (e.g. ‘Stay Safe’, ‘Stay On Path’) (*n* = 11), brochures/pamphlets (*n* = 8), Posters (*n* = 8), and T.V/radio (*n* = 2).

Survey respondents were also asked from whom they would like to receive risk information. The most selected option was from ‘official national parks organizations’ (*n* = 34, 68%), followed by ‘friends and family’ (*n* = 19, 38%). Social media platforms directly (e.g. Instagram alerts) and government sponsored advice on social media were both chosen 13 times, with ‘influencers’ receiving a count of 5 and ‘other’ 1.

### Ad recall from survey data collection

These results assess whether participants recognized any of the campaign materials.

Respondents were asked if they could recall seeing any of six images that had been posted on Instagram in the previous week and were allowed to recall more than one of the images. A third (32%; *n* = 16) recalled seeing a post on Instagram. The most recalled post was Post B (14 times) (Supplementary File S4) followed by Post F and C which were each recalled four times. Post E was recalled three times. Post A was recalled two times. Post D was recalled one time.

Ad recall varied somewhat by age group. Among 25–34 year olds, 47% (*n* = 9/19) recalled at least one campaign post, compared with 23% (*n* = 3/13) of 18–24 year olds, 17% (*n* = 2/12) of 35–44 year olds, and 33% (*n* = 2/6) of 45–54 year olds. These differences should be interpreted cautiously given the small sample size.

It is important to note that recall rates are based on the entire sample (*N* = 50), though only 56% (*n* = 28) reported Instagram as their primary social media platform. Thus, actual recall among Instagram users specifically may be underestimated, and these figures should be interpreted with this limitation in mind.

### Content analysis of comments

Fifty-five comments received by Instagram users were analysed in response to both paid advertisements and an organic in-feed post (Table 3). The analysis aimed to capture audience reaction, key discussion points, and areas for improvement in risk communication messaging.

**Table 3.** Themes identified in content analysis of user comments on communication campaign posts.

Theme	Responses *N* (%)	Key finding	Example response
Criticism of post and tone	13 (24%)	Users expressed dissatisfaction with how the message was framed rather than the message itself. Comments often described the tone as ‘passive-aggressive, condescending, or unnecessarily critical of visitors behaviour for photography’.	‘Your post came across very passive-aggressive. FYI. And that wasn’t the message that people are reading. I would love for you guys to start doing more workshops or collaborations with local artists or photographers that can provide you guys with that exposure and education all in one’.
Frustration with messaging	11 (20%)	‘Contained sarcastic replies and suggested the campaign had failed to resonate with some users’.	‘Thanks for the tip guys, I’ll just post photos of rangers looking at trees and at off-track lookouts to advertise our beautiful parks. Got it’.
Agreement with message	11 (20%)	Several users acknowledging the importance of addressing unsafe influencer behaviour and supporting the campaign’s intent.	‘people please follow the signs!’
Influencer criticism and authenticity	**3** (**5)**	Highlighted concerns about social media influencers ‘encouraging unsafe behaviours’ and setting unrealistic expectations for national park visits.	‘I see so many ‘influencers’ risking injury just to get the best shot’.
Photography and artistic expression	**3** (**5)**	Reflected discussions about ‘the role of creativity and digital editing in nature photography’, with some users feeling that the campaign unfairly criticized artistic interpretations of landscapes	“It is most likely influenced by their environmental surroundings and creative mind in what they see. This allows people to become artistic individuals within such wonders of nature—Art is an expression of one’s views—and should be celebrated as such.”
Park management and visitor experience	**3** (**5)**	Contained comments focused on ‘broader park policies’ and visitor expectations rather than the campaign itself.	“Time in nature makes space for life’s best moments, so get out there, be safe and enjoy them.”
Other	**17** (**31)**	Included ‘unrelated remarks, humour, and general engagement’, indicating varying audience reactions beyond the campaign’s intended discussion points.	‘Queensland National Parks what is a ‘travel inspo”?’

Comments were classified into seven key themes: frustration with messaging; agreement with message; criticism of post tone; influencer criticism and authenticity; photography and artistic expression; park management and visitor experience; and other miscellaneous responses. The content analysis showed a polarized response to the campaign. The largest categories were criticism of post tone (24%) and frustration with messaging (20%), with many users describing the campaign as passive-aggressive, condescending, or sarcastic. Conversely, agreement with the message (20%) demonstrated that some participants supported the intent of discouraging unsafe behaviours. Smaller proportions of comments highlighted influencer criticism and authenticity (5%), photography and artistic expression (5%), or park management and visitor experience (5%). Nearly a third of comments (31%) were coded as Other, which largely comprised unrelated humour, short remarks, or off-topic engagement that did not add substantive insights into risk perceptions.

A breakdown of these themes and representative comments is provided in Table 3.

## DISCUSSION

This study presents a process and impact evaluation of a co-produced social media risk communication campaign, developed in partnership with a national park authority and guided by the TPB. The campaign aimed to reach social media users visiting aquatic locations and influence their safety-related decision-making, countering the growing trend of influencers and visitors engaging in and promoting risky behaviours in pursuit of ‘Insta-worthy’ content (Bozzi 2020, Goh 2020, Cornell *et al*. 2024b).

This study triangulated data from both digital and in-person sources, including 50 on-site surveys conducted with visitors to a Queensland national park and user engagement metrics from the social media campaign. Despite a modest budget, the campaign reached an online audience of over 1.1 million people, demonstrating the potential of targeted social media communication to influence visitor behaviour in national parks (Ghahramani *et al*. 2022). The landing page traffic supports the idea that Instagram can function as a gateway to more detailed official safety content, reinforcing the value of integrated platform strategies in digital risk communication (Evans *et al*. 2022).

The campaign’s reach and engagement indicate a positive response to messages aligned with TPB constructs (Ajzen 1991). In interpreting campaign performance, it is important to note that we did not measure behavioural outcomes, but rather message recall and audience perceptions. Messages that emphasized personal risk and included direct safety appeals were the most frequently recalled by participants and were often described as useful or impactful in open-text responses. This suggests that clear, safety-oriented framing may be more memorable for audiences, though further testing would be required to determine whether such approaches influence actual behaviour.

In addition, recall patterns varied across demographic groups, with the highest recall observed among 25–34 year olds compared to lower levels among younger (18–24) and older visitors. Although these differences should be interpreted cautiously due to the small sample size, they suggest that tailoring campaign messaging by age group may enhance resonance and effectiveness. For example, younger visitors may benefit from more visually dynamic formats such as short-form Reels or influencer-led content, while older visitors may engage better with direct, information-rich messaging. Future campaigns should therefore consider demographic targeting alongside overall message design to optimize reach and impact.

Content analysis of comments showed evidence of attitude shifts and endorsement of safety norms (Bosnjak *et al*. 2020), while actionable advice was well received by some participants, suggesting improved perceived behavioural control (Darker *et al*. 2010). However, tone emerged as a key issue, with mixed reactions pointing to the need for future calibration (Gough *et al*. 2017).

These polarized responses reflect a tension between engagement tone and perceived authority. While many appreciated direct messaging, others found it condescending, reinforcing that even well-designed content must be audience-tested for tone and framing (Taba *et al*. 2023). Although this study’s materials were co-produced with a land management authority and informed by social media influencer interviews, user content analysis of comments revealed a disconnect between the intended message and audience reception. Specifically, when a lighter tone was used in an organic post, users perceived a different central issue than what the risk communication materials aimed to convey (Cornell *et al*. 2024b). Content analysis indicated that nearly half of the comments expressed frustration or a negative response towards the communication materials.

To improve audience reception and effectiveness, we recommend integrating TPB principles (Ajzen 1991) more explicitly into future social media campaigns. This includes shaping attitudes by clearly demonstrating the dangers of ignoring warning signs, influencing subjective norms by leveraging respected community figures, and enhancing perceived behavioural control by providing practical and actionable safety guidance. Our findings highlight the importance of aligning risk communication strategies with user preferences and behaviours to maximize impact.

Results from in-person surveys indicate that social media may play a role in influencing visitation patterns. Surveys revealed that a third of visitors attributed their visit to seeing content on social media. Analysis revealed that visiting the park with the specific intention of taking photographs was significantly associated with participants attributing their visit to social media. As a result, land managers may consider prioritizing communicating risks to those social media users who wish to visit national parks specifically to take photographs (Cornell *et al*. 2023). These findings are in line with previous research suggesting that national parks visitation has been increasing due to photography and social media instigated sight-seeing (Wichman 2024). Land managers have attested to a change in visitor behaviour on their parks and purported that this change is driven by social media and tourists’ search for ‘Instagrammable’ and photogenic locations, that sometimes put them in situations of increased risk (Cornell *et al*. 2024b). Research has illustrated that many land management organizations are ill equipped to deal with the rise of this new kind of tourism (Cornell *et al*. 2024b). Therefore, the present research is the first step in aiming to address the gap in knowledge that land managers face in communicating risks to social media-driven visitors.

While this study focused on aquatic safety within a national park, the findings have broader relevance. Increasingly, social media is drawing visitors to unpatrolled or high-risk waterways outside formal park boundaries, such as remote beaches, creeks, or waterfalls (Cornell *et al*. 2025). The same dynamics observed here—visually curated content obscuring hazards, and visitors seeking ‘Instagrammable’ experiences—may also apply in these contexts.

These insights have practical implications for land managers and policymakers. To mitigate the risks associated with social media-driven visitation, it is crucial to involve social media users and influencers in the process of designing risk communication materials. This collaborative approach can ensure that the messages are engaging and resonate with the intended audience, ultimately promoting safer behaviours and enhancing visitor safety in national parks. By aligning messaging with user expectations and behaviours, land managers and policymakers can more effectively promote responsible park use and minimise potential hazards associated with social media-driven visitation.

### Recommendations for future risk communication campaigns on social media

Our findings suggest that message tone should be explicitly tested alongside content in future campaign development, as resonance is shaped not only by what is said but how it is said. The findings suggest that risk communication campaigns must balance authority with relatability, particularly on platforms like Instagram, where users are sensitive to perceived top-down messaging (Evans *et al*. 2022).

Although this study used data and insight gained from previous research with land managers (Cornell *et al*. 2024b), influencers, and social media users (Cornell and Peden 2025), the current study did not directly co-design the communication campaign with the targeted audience. Future campaigns should include the targeted demographic in the co-production process, if feasible, as previous research has shown effectiveness of this methodology in the field of water safety and injury prevention (Koon *et al*. 2023).

While not possible in the current study, collecting data on actual behaviour would provide valuable insights. Future studies should consider measuring actual behaviours (e.g. adherence to safety guidelines) to strengthen understanding of campaign impact (Saunders *et al*. 2019). Furthermore, evaluating such a communication campaign across different national parks and natural settings will improve understanding of effectiveness in various contexts and environments, and among potentially different cohorts of visitors.

The study highlights the importance of integrating digital and physical information sources to reinforce safety messages. Future campaigns should ensure that consistent messaging is provided through on-site signage, which should be clear and visually engaging, placed at key locations within national parks to remind visitors of safety guidelines. Official websites should offer comprehensive information, easily accessible to visitors planning their trips. Additionally, continuous engagement with visitors through social media posts, stories, and ads should leverage the interactive features of these platforms.

The dynamic nature of social media requires ongoing monitoring and adaptation of communication strategies (Cornell *et al*. 2024c). Future efforts should include the use of real-time analytics to track engagement, reach, and sentiment, allowing for quick adjustments to the campaign. Regularly soliciting feedback from users in an iterative manner will help identify areas for improvement and ensure the messaging remains relevant and effective. Implementing a cycle of continuous improvement, where each campaign builds on the insights and lessons learned from previous efforts, will contribute to the development of more effective social media risk communication campaigns.

### Strengths and limitations

A key strength of the study was the participatory, co-produced campaign approach, which was informed by both park authorities and the target audience in formative design and the target audience directly in evaluation.

Inclement weather proved a significant challenge during Phase 3. Unfortunately, surveys were only successfully collected from one of the two planned sites. The lead author also attended Josephine Falls in Wooroonooran National Park, Far North Queensland, to collect data, but due to severe and torrential rain over the data collection period, parks were closed and no surveys were able to be collected. The influence of social media on visitation patterns should be interpreted with caution. The total number of visitors to Curtis Falls during the survey weekend was not officially recorded by QPWS limiting our ability to calculate an exact response rate or estimate representativeness relative to the broader visitor population.

Although statistical tests suggest an association, the small sample size and reliance on self-reported data introduce potential biases and limit the generalizability of this finding. However, the insights gained are useful for future research and the authors are unaware of similar research having taken place.

While the sample size (*n* = 50) of those surveyed is relatively small, it aligns with prior pilot studies in behavioural research, providing preliminary insights into social media-driven risk perception and engagement. For instance, a study investigating the relationship between social media use and loneliness among rural youth in New South Wales involved 47 participants aged 16–24 (Gregory *et al*. 2023). These examples illustrate that smaller sample sizes are common in pilot studies, especially when exploring new or specific behavioural phenomenon related to social media use.

## CONCLUSION

This study demonstrates the potential for delivering co-produced safety messaging through social media platforms in a national park context. It highlights the dual role of social media as both a driver of increased visitation and a potential tool for effective risk communication. Land managers may be able to better address the challenges posed by social media-influenced visitation and ensure the safety and enjoyment of all visitors to national parks by leveraging the reach of social media and incorporating user feedback.

## Supplementary Material

daaf181_Supplementary_Data

## Data Availability

Data pertaining to the surveys is available upon reasonable request. Social media data used for this study is owned by Queensland Parks and Wildlife Service and may be available upon request.
