# Supplementary material for: Design and evaluation of a co-produced social media campaign to promote aquatic safety in Queensland national parks
Source: Health Promot Int. 2025 Oct 30;40(6):daaf181. doi: 10.1093/heapro/daaf181 (PMC12574670; doi:10.1093/heapro/daaf181)
Supplement: daaf181_Supplementary_Data [file daaf181_supplementary_data.zip › Supplementary File 1..docx]

| **Data Source** | **Key Findings** |
| --- | --- |
| Social media user survey (n=509) | - Instagram most frequently used for trip planning (68%) - Preference for safety info to come from official park sources - Tone should be casual, visually engaging, and non-authoritarian |
| Influencer interviews (n=18) | - Influencers did not feel responsible for follower risk-taking - Expected land managers to take a greater role in setting expectations - Acknowledged risk-taking behaviour in followers motivated by aesthetics or content replication |

***Supplementary Table 1. Summary of formative findings used to inform campaign design***
