# Supplementary material for: Design and evaluation of a co-produced social media campaign to promote aquatic safety in Queensland national parks
Source: Health Promot Int. 2025 Oct 30;40(6):daaf181. doi: 10.1093/heapro/daaf181 (PMC12574670; doi:10.1093/heapro/daaf181)
Supplement: daaf181_Supplementary_Data [file daaf181_supplementary_data.zip › Supplementary File 2.docx]

| **Landing Page** | **Target Region** | **Key Messages** | **TPB Elements Emphasised** |
| --- | --- | --- | --- |
| Statewide | All of QLD | Filter realism, safety signage, authentic content | Attitudes, Subjective norms, Control |
| SEQ | South East QLD | Overcrowding, water risks, alternate activities | Control, Attitudes, Realistic planning |

***Supplementary Table 2. Summary of landing page key messages and relation to TPB elements.***
