# Supplementary material for: Design and evaluation of a co-produced social media campaign to promote aquatic safety in Queensland national parks
Source: Health Promot Int. 2025 Oct 30;40(6):daaf181. doi: 10.1093/heapro/daaf181 (PMC12574670; doi:10.1093/heapro/daaf181)
Supplement: daaf181_Supplementary_Data [file daaf181_supplementary_data.zip › Supplementary File 3.docx]

| **Codebook Category** | **TPB / Communication Alignment** |
| --- | --- |
| Frustration with message | Negative attitude toward the message |
| Agreement with message | Positive attitude |
| Influencer Criticism and Authenticity | Subjective norms / attitudes about social norms |
| Photography and Artistic Expression | Perceived behavioural norms, message rejection |
| Park Management and Visitor Experience | Environmental context, perceived control or expectations |
| Criticism of Post and Tone | Message reception / tone acceptance |
| Other | Catch-all category (good to have — should be defined) |

***Supplementary Table 3. Codebook categories used in Instagram content analysis of comments and their alignment with behavioural theory constructs.***
