# Supplementary figures and images for: Design and evaluation of a co-produced social media campaign to promote aquatic safety in Queensland national parks
Source: Health Promot Int. 2025 Oct 30;40(6):daaf181. doi: 10.1093/heapro/daaf181 (PMC12574670; doi:10.1093/heapro/daaf181)

**
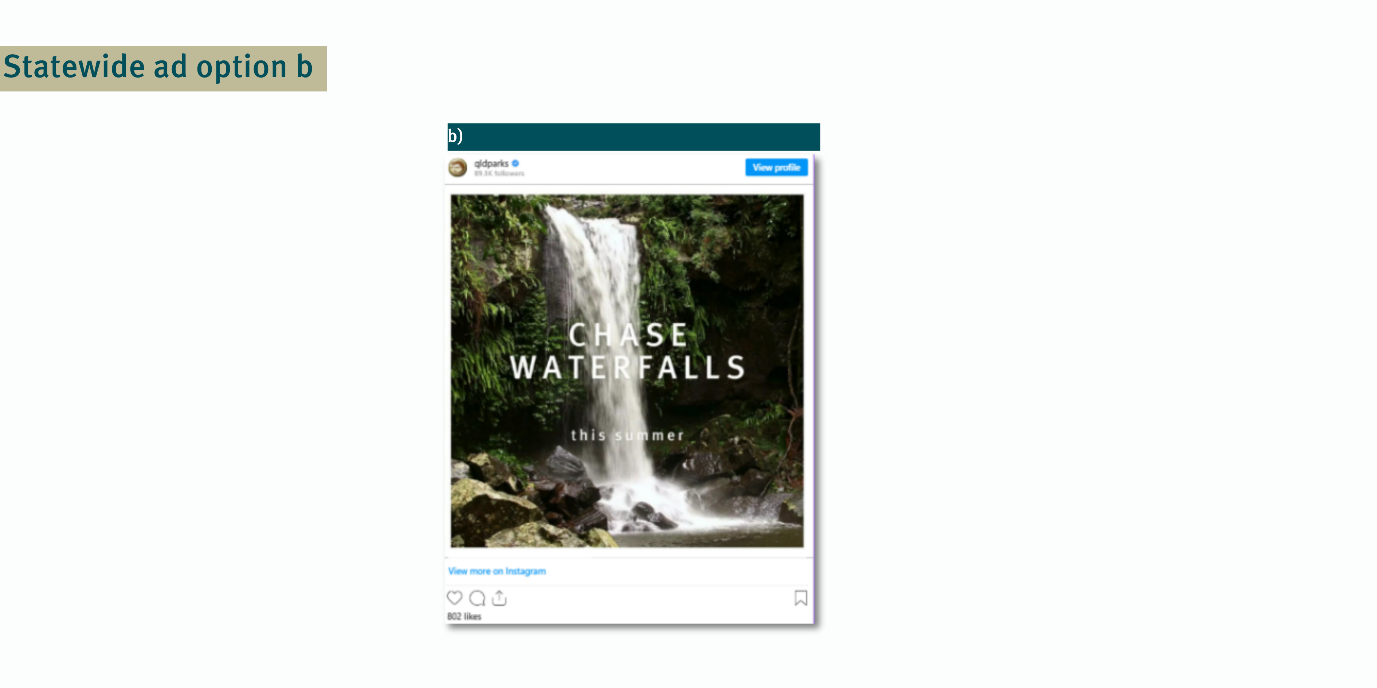
**

Supplement: daaf181_Supplementary_Data [file daaf181_supplementary_data.zip › Supplementary File 4.docx]
